# Body image and psychosocial well-being among UK military personnel and veterans who sustained appearance-altering conflict injuries

**DOI:** 10.1080/08995605.2022.2058302

**Published:** 2022-05-03

**Authors:** Mary Keeling, Heidi Williamson, Victoria S. Williams, James Kiff, Sarah Evans, Dominic Murphy, Diana Harcourt

**Affiliations:** a Centre for Appearance Research, Department of Social Sciences, Faculty of Health and Applied Sciences, University of the West of England, Bristol, England; b Combat Stress & King’s Centre for Military Health Research, King’s College London, London, UK

**Keywords:** Combat injuries, military veterans, body image, limb-loss, scarring

## Abstract

A modest but significant number of military personnel sustained injuries during deployments resulting in an altered-appearance (e.g., limb loss and/or scarring). Civilian research indicates that appearance-altering injuries can affect psychosocial wellbeing, yet little is known about the impact of such injuries among injured personnel. This study aimed to understand the psychosocial impact of appearance-altering injuries and possible support needs among UK military personnel and veterans. Semi-structured interviews with 23 military participants who sustained appearance-altering injuries during deployments or training since 1969 were conducted. The interviews were analyzed using reflexive thematic analysis, identifying six master themes. These themes indicate that in the context of broader recovery experiences, military personnel and veterans experience a variety of psychosocial difficulties related to their changed appearance. While some of these are consistent with evidence from civilians, military-related nuances in the challenges, protective experiences, coping approaches, and preferences for support are evident. Personnel and veterans with appearance-altering injuries may require specific support for adjusting to their changed appearance and related difficulties. However, barriers to acknowledging appearance concerns were identified. Implications for support provision and future research are discussed.

**What is the public significance of this article?—**This study highlights the psychosocial experiences and unmet support needs of personnel and veterans who sustained appearance-altering injuries (e.g., scarring and limb loss). Findings can inform the development of interventions to support adjustment following appearance-altering injuries.

The nature of military service means some personnel, especially those serving in combat roles during wartime, sustain injuries resulting in changes to their appearance (e.g., physical scars, limb-loss). Between April 2005 and March 2020, 10,100 UK service personnel and ex-personnel (veterans) were in receipt of Armed Forces Compensation due to “injury, wounds and scarring” (Ministry of Defence, 2020b) and 336 UK personnel sustained injuries that included traumatic or surgical amputation of one or more limbs (Ministry of Defence, 2020a). In the US between October 2001 and June 2015, 1,645 personnel sustained major or partial limb/digit amputations due to battle-related injury (Fischer, 2015).

Research with civilians indicates that having burn injuries or limb-loss can increase body image dissatisfaction (BID), which may be associated with depression, general anxiety, low self-esteem and low quality of life (Cleary et al., 2020; Holzer et al., 2014). Moreover, difficulties adjusting to an enforced change to appearance may affect the ability to recognize the self, which can disrupt the self-concept (Wisely & Gaskell, 2012). People who look noticeably different from culturally sanctioned appearance ideals can also experience negative public reactions, social stigma and social rejection. Name-calling, staring, unsolicited questioning from strangers and being avoided by others, have been reported among adults with scarring from burn injuries and limb-loss (Horgan & MacLachlan, 2004; Martin et al., 2017). Fear of being negatively judged by others because of an altered or unusual appearance, can lead to social anxiety (Clarke et al., 2014; Rumsey & Harcourt, 2012), resulting in social avoidance, isolation and behaviors to conceal scars or prosthetics (Holzer et al., 2014; Levine et al., 2005). In addition, evidence indicates that trauma symptoms related to the injury may be associated with appearance concerns (Shepherd, 2015).

While existing evidence highlights the psychosocial challenges of adjusting to a changed appearance following burn injuries and limb-loss among civilians, there is a dearth of research into the experiences of military personnel and veterans. A recent literature review (Keeling et al., 2020) to determine psychosocial experiences, body image concerns and associated support needs among military personnel and veterans with combat-related appearance-altering injuries (e.g., traumatic limb loss, burn scarring, shrapnel scarring, scarring from gunshot wounds, caused during military operations), identified just four published studies: three from the US and one from Turkey. This small collection of studies provides emerging evidence that much like civilians, veterans with appearance-altering injuries may experience BID and associated symptoms of depression (Akyol et al., 2013; Weaver et al., 2014) and lower quality of life (Akyol et al., 2013), as well as challenges adjusting to the external reality of their changed body (Cater, 2012; Messinger, 2009). Veterans also report concerns around public stigma, appearance-related self-consciousness, social avoidance and isolation (Cater, 2012; Messinger, 2009). Although a direct association between Post Traumatic Stress Disorder (PTSD) and BID was not found, Weaver et al. (2014) suggest that scars may function as a reminder of the injury, which may affect BID and trauma symptoms, but that this requires further investigation (Weaver et al., 2014).

Although consistencies between civilians and veterans are apparent, Cater’s (2012) research with female veterans with limb-loss indicates that military culture plays a role in recovery. Women veterans attributed their ability to adjust to their changed appearance and overcome anxieties related to public stigma and diminished self-confidence, to the military ethos of hardiness and strength. They reported being proud to be seen with their prosthetic limbs, considering them a badge of honor representing service to their country. Cultivating a positive outlook and positively reframing their injury experiences, as well as recovering among others with similar injuries, were also reported to help (Cater, 2012). Although evidence of masculinity in military culture and the impact on body ideals was not directly examined in Weaver et al. (2014), they do suggest that masculine body ideals of muscularity may have implications for how injured male veterans experience their changed appearance.

On joining the Armed Forces, the civilian body is incorporated into the military through the practices of training. Having a strong, capable, muscular, physically able body is a non-negotiable requirement of military service (Godfrey et al., 2012), and appearance-altering injuries may represent a loss of the military body (Keeling & Sharratt, 2022; Wool, 2015). Additionally, and further affirmation of the importance of the military body, serious physical injuries typically result in medical discharge and the termination of a military career. As stated above, adjusting to a changed appearance likely disrupts the self-concept (Wisely & Gaskell, 2012). In the military context, this disruption of the self may be exacerbated by the coinciding loss of a military career and associated military identity (Keeling, 2018).

Despite emerging evidence indicating that veterans with appearance-altering injuries may experience psychosocial challenges and that cultural nuances may influence their recovery and adjustment, a deeper understanding of the experiences of military personnel and veterans who sustained appearance-altering combat-injuries, and their support needs, is needed. This study therefore aimed to gain experiential insights into the body image, psychosocial and appearance-related experiences of UK military personnel and veterans who had sustained appearance-altering injuries during deployments or deployment training, any support they may have received, and any unmet support needs.

## Method

### Design

Based on the study’s experiential focus, a qualitative approach was deemed most appropriate, using semi-structured interviews and a Reflexive Thematic Analysis (TA; Braun & Clarke, 2019) methodological approach.

### Participants and recruitment

Participants were UK Armed Forces serving personnel or veterans with permanent appearance-altering injuries (e.g., scarring, limb loss) sustained during operational deployment or training for deployment since 1969 (to be inclusive of those injured in modern combat since “The Troubles” in Northern Ireland, the Falklands conflict and first Gulf War). Injury must have occurred at least one year prior to interview to exclude those undergoing acute medical recovery and rehabilitation. Veterans were recruited (and interviewed) between March and September 2019, serving personnel between March and November 2020. Study advertisements and invitation letters were shared with potential participants via UK military third sector support organizations, social media (Twitter, Facebook, and Instagram), and via snowballing (e.g., those who participated were invited to share the study invitation and advertisement with other veterans who they knew, who might be interested and eligible to participate). Interested parties contacted the study team via e-mail or telephone, were provided a participant information sheet, and asked to complete an eligibility screening questionnaire (including asking for the participants’ service number to confirm military service status; to describe their injury, for example, “lower right below knee limb loss”; about the context of how the injury was sustained, for example, “in a tank during patrol in Afghanistan,” and the year they were injured). Those eligible provided written informed consent via e-mail or post and suggested a suitable time to be interviewed.

### Procedure and materials

Individual telephone interviews were conducted by the lead author, guided by a semi-structured interview schedule. The semi-structured approach facilitated flexibility; the order of questions asked was not prescriptive and the interviews were instead shaped by participants. The schedule covered questions about: military background and context of injury; their changed appearance; adjusting to these changes; acceptance; and, appearance-specific support. Interviews lasted between 50 and 90 minutes. Participants were offered a £20 online shopping voucher to thank them for participation. Interviews were audio recorded and transcribed verbatim by the third and fifth authors.

Patient and Public Involvement (PPI) feedback on the feasibility and acceptability of the study advert, invitation, and interview schedule was provided by the study’s Advisory Group (AG; veterans with lived experience of appearance-altering injuries). This led to changes in the advert layout, invitation length, language use, and phrasing of interview questions.

Ethics approval was gained from the University of the West of England, Bristol, research ethics committee (ref: HAS.19.01.113) and the Ministry of Defense ethics committee (ref: 1014/MODREC/19).

### Sample

The sample consisted of 23 participants; 20 veterans and three serving personnel. The smaller number of serving personnel reflects the typical nature of appearance-altering conflict-injuries often necessitating medical discharge. Further participant details are in Table 1.

**Table 1.** Sample characteristics.

Sociodemographic Characteristics		Injury Characteristics	
*Gender*		*Type of injury*	
Female	1	Physical scarring*	14
Male	22	Traumatic limb loss	2
*Ethnicity*		Scarring & limb loss	7
Black Commonwealth^1^	1	*Type of limb loss*	
White British	21	Single lower limb	4
White Commonwealth	1	Double lower limb	4
*Age* mean (range)	38.48 (32–53)	Double lower & single upper	1
Military Characteristics		*Years since injury* mean (range)	10.22 (5–27)
*Serving status*		*Cause of injury*	
Serving	3	Direct enemy action**	19
Left service (veteran)	20	Fall (on deployment)	1
*Service branch*		Training accident (non-deployment)	3
Naval Services	1	*Location injury sustained*	
Army	21	Afghanistan	17
Royal Air Force	1	Iraq	2
*Engagement Type*		Northern Ireland	1
Regular	21	Training exercise in the UK	3
Reserve^2^	2	*Time period injury sustained*	
*Years military service* mean (range)	11.78 (4–26)	Pre 9/11	1
*Years since discharge* mean (range)	6.15 (1−26)	Post 9/11	22

^1^Members of the 54 Commonwealth Nations are able to apply to join the British Armed Forces; ^2^British Armed Forces Reserves are civilian volunteers who serve in the military part-time, routinely undergoing training and military operations alongside Regular personnel who are full-time paid military personnel; *1 severe burns; **E.g., Improvised Explosive Device (IED), gunshots, explosions and Rocket-propelled grenades (RPG).

A sample of n = 23 was deemed a suitable number of participants to allow for exploration of a narrow topic (Malterud et al., 2016; Morse, 2000). In addition, as this study took a critical realist and exploratory stance, the researchers did not intend to provide a “complete” description of participants’ experiences (Malterud et al., 2016).

### Researcher positioning and reflection

In reflexive TA, researcher subjectivity is conceptualized as a resource for knowledge production; transparency of researcher positioning and engagement in reflexive practice is vital (Braun & Clarke, 2020). All authors are white British and nonmilitary. Five are females and two are male. Four of the authors’ fathers had served in the military and one author also had siblings who had served. None of the authors’ fathers or siblings who served had sustained service-related injuries and none of the authors have an appearance-altering injury. Before the interviews, the first author reflected on their assumptions about the participants and possible research outcomes. The authors reflected on their position as “outsiders” and how it might influence their understanding and interpretation. AG advisors were consulted about the interpretation of the results. This allowed “insiders” to consider the analysis and provide insight from their contextual and situational position.

### Data analysis

Reflexive TA is a qualitative methodology used to identify and interpret patterns or themes of shared meaning within the data (Braun & Clarke, 2019), facilitating a deeper understanding of participants’ experiences. An inductive critical realist approach was taken, with analysis conducted at a latent level. Veterans’ transcripts were analyzed first as at this time (October 2019) it was unclear if interviews with serving personnel would be possible. To check for transcript accuracy and aid data familiarization, the first author read each transcript while listening to the recording. A line-by-line examination of the data then led to the identification of codes. Once coding was complete, the first and third author considered the codes, examining similarities and differences, how some were naturally grouped and others demonstrated opposing or contrasting experiences. This led to the generation of nine master themes with sub-themes. These candidate master and sub-themes were discussed with the wider team, leading to the merging of some master and sub-themes to capture the participants’ experiences. This draft was presented to the AG who reported that the master and sub-themes resonated with their experiences, although minor changes to language used to describe themes were provided.

A year later, when interviews with three serving personnel had been conducted and transcribed, the first author analyzed the new transcripts, following the same process. The codes were considered against the veterans’ master themes and sub-themes. Serving personnel codes were consistent with the veteran themes and sub-themes. All codes were incorporated, but not all sub-themes were represented in the serving personnel transcripts. This is noted in the results where a theme is introduced as representative of the veterans’ experiences only.

## Results

Reflexive TA led to the generation of six master themes with sub-themes that represent appearance-related challenges, coping mechanisms, and experiences of support (Table 2). Of note, while appearance was the focus, the participants’ appearance-related experiences are situated within the context of their broader recovery experiences following life-changing physical combat-injuries. This broader context included: experiences of medical treatment and rehabilitation; the interaction of physical and mental health; the impact on their lives, especially changes to family roles and their military career; the military ethos of hardiness and strength; and, for those medically discharged, their transition to civilian life. The detail of this context is not expanded on here since it is not the focus of this specific research but should be considered when interpreting these results. To address the research aims, the six appearance-specific master themes and subthemes are presented alongside supporting verbatim quotes, with participant pseudonyms.

**Table 2.** Master themes and sub-themes.

Master themes	Sub-themes
Appearance related challenges	Intrusion, stigma and feeling different.
	Loss of military physique and fitness.
	Scars as a reminder.
	Romantic relationships and intimacy.
	Social impact on children.
Psychological impact ofappearance challenges	Increased salience of appearance.
	Appearance-related distress.
	Accepting and adjusting to how I looknow.
	Co-morbid mental health difficulties.
Protective experiences	Expecting to be injured.
	Injured veteran peer support.
	Being seen as an injured war veteran.
	Being in spaces where looking different isnormalized.
	Accepted and protected by significantothers.
Cognitive coping	Making meaning of scars.
	Rationalizing enacted stigma.
	Downplaying the importance ofappearance.
	Accepting what you can’t change.
	Optimistic outlook.
	“It could have been worse.”
Active coping	Rebuilding self.
	Recovery via sporting activities.
	Creating a safe narrative to manage others.
	Making it work for me.
	Avoidance.
Appearance support, delivery,and barriers	Obstacles to acknowledging appearance.
	Appearance support we’d like.
	Barriers to care.
	Delivery preferences.

### Appearance-related challenges

All participants experienced challenges related to their changed appearance as represented by five sub-themes: Intrusion, stigma and feeling different; Loss of military physique and fitness; Scars as a reminder; Romantic relationships and intimacy; and Impact on children. These sub-themes highlight the multiple ways in which adjusting to a changed appearance can affect the individual, their relationships, and their children. The degree of concern, personal significance and comfort discussing these challenges varied between participants.

#### Intrusion, stigma, and feeling different

Experiencing public intrusion, such as people asking questions, staring, taking photos and making insulting comments, was reported by all participants. For some, this led to feeling a depleted sense of social anonymity and many reported that family and close friends were also subject to these intrusions. Several participants made comparisons between children and adults, differentiating children as curious and adults as judgmental.

So, um asking questions, looking, asking to touch, it’s just all of these things … And they ask all of these probing questions which I find a bit inappropriate (John, serving).

… you know kids, young kids are just so innocent and, they’re inquisitive, and parents are like, “don’t look don’t look”, they’ll you know, look like embarrassed or, they give the vibe that, you know, it’s a negative, if that makes sense? (Alice).

Certain aspects of an altered appearance (e.g., scars on the face, missing fingers, asymmetrical or “ugly and untidy” rather than “neat” scars) were experienced as drawing more attention or feelings of judgment, and/or created feelings of being “different.” Among some who had lost limbs, clothing restrictions exacerbated their feelings of looking different. For some participants, the impact of social stigma associated with looking different on future employment was an additional concern.

So, you know, if you were going to a formal event and there’s a dress code you like to fit in with that dress code … it still makes me feel uncomfortable when you’re you know maybe you’re at a smart event and every single other person is in a suit … So you know these problems come in and sort of plague you (Arron).

What are my chances of even getting a job that would you know, give me what I want if I um, if I, you know, if I was to try and go to interviews and I turned up in a wheelchair, you know (Daniel, serving).

#### Loss of military physique and fitness

Nine participants, including all three serving personnel, made self-critical comments about their post-injury physique; some directly related to their changed appearance, others related to weight gain caused by inactivity during recovery or periods spent in wheelchairs. The realization of weight gain often led to efforts to attain a more personally and socially acceptable physique. The loss of their previous muscular and toned bodies (replaced with weight gain and reduced muscle definition) affected confidence, body image and their sense of dignity and masculinity. Apparent teasing, indicated in the quote from John, highlights the standards of physique expected within the military and the possibility of receiving “banter” if deviating from that ideal.

And then you start putting weight on and then you put too much weight on *laughs* … that’s never a good look. A sort of just a blob with limbs missing. Er and then you start sort of trying to get under control of how you should be eating and you should be training and stuff … (Mark).

… my pectoral muscles droop slightly. Er, they go over the scar tissue which really just makes you like, someone pinched it, one of the lads pinched it the other day and went “oh yeah put on some weight.” And I thought oh, cheers. I’m literally being sexually groped, by, like this is how women who get, you know, sexually assaulted at train stations must feel. You know I was like *laughs* Oh my god, this guy actually just touched my breast *laughs*. Like, yeah gave it, a proper little squeeze as well. I was fucking heartbroken (John, serving).

#### Scars as a reminder

For five of the veterans and two of the service personnel, their scars, walking aids, and prosthetics, acted as a reminder of their injury and associated trauma, guilt, and negative experiences. For some, the constant reminder restricted their ability to move on, reflecting their difficulties accepting events. In contrast, one of the serving personnel experienced the reminder positively, indicating acceptance of their injury. Of note, they had been injured during training where nobody else had been injured.

I’ve still not accepted it. So, um, but that’s got, you know and the scar tissue is a reminder of that, which is why I’ve decided to try and get it covered up (John, serving).

… in terms of, my appearance yeah sometimes I think, it’d be nice to … to not have it there but, I accept that, you know it’s a reminder of what happened. It’s just a part of my life. You know as it is a part of my appearance (Craig, serving).

#### Romantic relationships and intimacy

Eight participants (including two serving personnel) described how their changed appearance affected their romantic relationships and/or sexual intimacy. Some of their partners did not like the appearance or feel of their scars, and the change to appearance affected some participants’ self-confidence, impacting their relationship and sexual intimacy. Practical challenges resulting from changes such as limb loss, created feelings of uncertainty in how to engage in sexual activities and frustrations due to physical limitations.

I generally sleep on the couch most of the time because yeah, the thought that she might feel a bit kinky and I dunno, I dunno what I’d do now you know? (Ethan).

Among single participants, knowing when to disclose their visible difference to potential new partners was challenging. Most overcame this by clearly displaying their difference, having their prosthetic visible to others or casually mentioning their scars early during a date. Some had sustained genital injuries, creating an additional challenge around disclosure. Genital injuries seemed to affect masculinity and confidence, and the risk of genital injury was widely feared with many of the blast-injured veterans reporting having immediately checked their genitals.

That’s quite tough obviously the injury that I mentioned earlier with my um in the nether regions *laughs* meat and two veg, that is a huge, that’s a massive impact on you a massive impact and it you know it’s a typical guy thing I’m sure um … you’ve lost something that makes you a man (Paul).

I said to my mate “don’t worry about my legs are my bollocks still there?” (Neil).

#### Social impact on children

For parents, “looking different” impacted their children. Children asked what was wrong with them, friends queried why their parent looked different, and some were bullied because of their parent’s appearance. Many interviewees found ways to manage these challenges, such as talking to their children about their injuries, but others were unsure of what to share, especially with younger children.

… and there was an incident where my oldest, he was probably about, 10 at the time I’m not quite sure. And he got picked on, for, his mum only having one leg and, we didn’t know about this until quite later on (Alice).

### Psychological impact of a changed appearance

Challenges adjusting to a changed appearance evidently affected psychological wellbeing, as reflected in four sub-themes: Increased salience of appearance; Appearance-related distress; Accepting and adjusting to how I look now; and Co-existing mental health difficulties.

#### Increased salience of appearance

For some participants, changes to how they looked increased the centrality of appearance in their self-concept and how they viewed themselves, with some considering themselves “disfigured.” For those whose appearance was central to their self-concept prior to injury, their changed appearance was distressing, with some now seeing themselves as unattractive compared to their previous “good looks.”

When it first happened, I did think that well I was disfigured you know … yeah, I think the initial thing was yes I, I am I have been disfigured (Simon).

I was quite a handsome young lad … I was a proud Paratrooper and that all changed after I got blown up … (Benjamin).

#### Appearance-related distress

Seventeen participants, including all serving personnel, experienced psychological distress related to their appearance such as feeling less attractive, feelings of disgust, shame, low self-worth, low self-confidence, and heightened self-consciousness. Some described their injuries in de-humanizing ways and for many their distress was compounded by social intrusion and stigma.

I just heard him turn round to his Mrs and be like “oh have you seen his leg? disgusting” and … that pretty much summed up how I felt (Patrick).

I felt like I looked like a hanging piece of meat in a butchers, if you know what I mean, and I felt that was a lot to do with my looks (Ethan).

#### Accepting and adjusting to how I look now

Nine of the veterans shared experiences of being surprised when catching a glimpse of themselves in a mirror or noticing others’ reactions toward them, as their internal image of their body still drew on the memory of their pre-injured appearance. This disconnect between their internal representation and the external reality of their appearance could be distressing and indicate that acceptance of their post injury appearance was an ongoing process.

You know I have no idea how long it takes for your brain to adjust to how it you know the picture that it has of itself. But I still clock myself, I’m surprised every now and then, and I catch it in a mirror … I still overestimate what I can do and what I look and you know I don’t know how long that takes to adjust but I’m not there yet (Arron).

#### Co-existing mental health difficulties

Thirteen of the participants, including one of the serving personnel, reported co-morbid mental health difficulties such as symptoms of depression, anxiety, post-traumatic stress, survivor guilt, substance use, and suicidal ideation. These difficulties seemed associated with the broader context of their injury, but likely interacted with the challenges and distress associated with their changed appearance.

The other boys didn’t make it so, and I you know, I had to live with it, and with the guilt of that, for quite a long time and I found it tough, it’s not been easy … I weren’t proud of being (pause) a survivor, I was really, I couldn’t talk about it for a long time (Benjamin).

### Protective experiences

The majority of participants shared experiences that were protective and/or created resilience as represented by five sub-themes: Expecting to be injured; Injured veteran peer support; Being seen as an injured war veteran; Being in spaces where looking different is normalized; and Accepted and protected by significant others.

#### Expecting to be injured

Six participants who sustained deployment-related injuries expected to be injured due to high rates of injuries and deaths in their deployed region, or because they understood that being injured is a risk of military service. Many believed their expectation helped recovery by enabling acceptance and a positive mind set. One veteran compared this with civilians who sustain similar injuries, suggesting their adjustment must take longer. None of the participants who sustained injuries during training reported the same expectation.

So, I guess if you’re a civilian and you’re you know I’ve got a friend of mine who was just out for a night out with his friend and got hit by a car and he had to wake up minus both his legs, but he was never in the situation where he expected anything like that to happen so the adjustment phase seems to be a lot more difficult (Arron).

#### Injured veteran peer support

The benefits of veteran peer support were reported by the majority (17/19) who sustained deployment-injuries, but not by those who sustained training injuries. Support from fellow injured personnel and veterans, and seeing others further along in their recovery, helped morale, goal setting, and motivation, and facilitated validation and feeling accepted. Peer support from others with similar injuries sustained in similar situations, was noted as unique to injured veterans compared to civilians. Many were also involved in formal programs, providing peer support to other injured veterans. Veteran peer support reflected a continuation of the military values of camaraderie, morale, and collectivism.

If you’ve got a problem the chances are one of them has already been through it or, if not, there’s a load of blokes that are willing to help you (Patrick).

#### Being seen as an injured war veteran

Due to their age, nature of injuries, and increased public awareness of injured military personnel, 12 participants reported feeling that the public often perceived them as injured war veterans. This was a positive experience for those who felt proud to have served their country and experienced their visible injuries as a “badge of honour,” for some alleviating concerns about being judged negatively.

I was proud to be able to like an amputee and because of my service, at that stage, so … I suppose, I kind of thought of like yeah as a like a badge of honour *laughs* (Charles).

In contrast, those experiencing survivor guilt or believing their injury was insignificant or illegitimate compared to veterans with “real” injuries, felt undeserving of the public’s positive regard and uncomfortable at the prospect of being considered a “hero.” For some, being “seen” as an injured veteran came with negative stereotypes such as having mental health problems or that they had committed dangerous and violent acts.

… comparing my injury to others ‘cos I feel a bit of injury guilt. Like you know I don’t deserve to be classed in this in the same … you’re calling these guys heroes and all this … and I didn’t feel I necessarily fell into that category (Neil).

… there’s this whole stigma of being a soldier and what did you do … and if you’re a trained killer … and then of course all of the sudden oh you’ve been injured so you must have psychological issues, this is their assumption of course (Edward).

#### Being in spaces where looking different is normalized

Most who sustained deployment-injuries recovered and rehabilitated in specific military facilities among others with similar injuries. Some veterans were employed in environments with similarly injured veterans and/or civilians. Being in spaces where “looking different” was “normal” alleviated challenges associated with having an unusual appearance, including intrusion and stigma. However, away from “protected” areas, the challenges of public spaces were experienced more intensely, especially upon initial discharge from the specific military rehabilitation center.

… but then, when you’re at Headley [military rehabilitation centre] you’re in that little Headley bubble and everyone there’s normal no matter what the injury and, once you’re away from that environment and you’re in with like real people it sometimes, you can have people can be negative to you indirectly and it can affect you (Alice).

#### Accepted and protected by significant others

Feeling that family, partners and friends accepted them following their injury was beneficial for nearly half of the veterans. Some partners explicitly stated that they valued the participant for who they were, not how they looked. For some, their friends had continued to treat them as they had prior to their injury, not making special exceptions or a “fuss.” Some veterans’ families showed protective behaviors, for example, in social situations where the veteran was experiencing discrimination or stigma.

My husband loves me for who I, you know, for who I am, um body parts and no body parts, he loves every wobbly bit of myself (Alice).

They’re like ‘well why don’t you just friggin ask him instead of asking us?’ or you know they’re straight back with a comment while I’ll just sit there or stand there (Ethan).

### Cognitive coping

Participants described how the way they thought about and framed their experiences had helped them cope, as demonstrated across six sub-themes: Making meaning of scars; Rationalizing enacted stigma; Downplaying the importance of appearance; Accepting what you can’t change; Optimism; and “It could have been worse.”

#### Making meaning of scars

A third of the participants, including two serving personnel, attached meanings to their scars, which cultivated a sense of control, mastery, and ownership. For example, interpreting their scars in terms of how they represented the different stages of their medical treatment; feeling good about their scars as a unique aspect of their life history; and considering them as indicators of masculinity, and looking cool. In contrast, those who had difficulties accepting their changed appearance, particularly those who experienced survivor guilt, attached negative meanings to their scars.

That scar’s associated with progress rather than negative memories and you know all that other stuff … the scar now is associated with forward progress and momentum and actually being able to carry forward into a more active lifestyle (Henry).

I kind of felt like I deserved to be punished um and I deserved the scarring coz of what happened to that young man (John, serving).

#### Rationalizing enacted stigma

Nine participants, including two serving personnel, coped with public intrusion and stigma by rationalizing such behavior. Using compassion and empathy, they interpreted people’s stares or questioning as inquisitive rather than judgmental, or considered that most people have not seen individuals with large scars, missing limbs and/or prosthetics before, or that they would have acted the same way if roles were reversed. Some participants attributed intrusive or stigmatizing behavior as the other person’s “problem.” These cognitive approaches seemed to minimize veterans’ internalization of intended or perceived judgment.

I just brush it off because yeah, it’s important to think these are people who’ve maybe never seen an amputee before or seen such a lot of scarring on people. And I was obviously the same. I never knew nothing about amputees or that until I’d actually been sent to Headley Court (Charles).

#### Downplaying the importance of appearance

Eight participants, including one serving personnel, demonstrated perspectives that seemed to minimize the value of appearance compared with other aspects of life, and focussing on what their body could do rather than how it looks. Being self-critical about appearance and wanting to look one’s best were normalized as something most people do, regardless of having a visibly different appearance. Normalizing appearance concerns served to downplay the importance of appearance in the context of their visible difference, minimizing appearance salience and potentially appearance-related distress.

So, I, you know, it was just all about, really it was all about what I could achieve, you know, what could I do, what does my body allow me to do. Um. As opposed to, ‘What did it look like?’ (Daniel).

#### Accepting what you can’t change

Resignation and accepting that staring and attention was inevitable seemed to help 11 of the veterans. To some extent, they appeared to free themselves from its impact and, for some, to accept it. Similarly, accepting that scars or limb loss were not things they could change meant they could accept their injuries and move on.

Instead of shying away from stuff you know I’m not gonna change what’s happened my legs not gonna grow back I’m not a gecko, so it sort of, accepts that you know that shit hit the fan, I’ve been through something, and, I’m not gonna change it nothing can change that so it’s sort of, learning to live with it and embrace it a little bit (Patrick).

#### Optimistic outlook

Eight veterans took an optimistic approach to their injuries and their appearance. This helped create motivation, hope, and a generally positive outlook and affect. Some felt their innate positive disposition aided their recovery, whereas others had deliberately cultivated an optimistic outlook upon realizing it would help.

I started thinking there’s light at the end of the tunnel, I can’t sit around feeling sorry for myself all my life coz it doesn’t get you anywhere (Benjamin).

#### “It could have been worse”

The majority of participants drew comparisons with others they perceived to have more severe injuries, and spoke about how their injury “could have been worse.” This downward comparison seemed to encourage a positive attitude toward life and recovery and, instead of taking a deficit-based view, could create a sense of gratitude.

I just happened to be the unfortunate bugger that come out of it, well, my mate, he come off a bit worse because he’s dead. You know, that could’ve been me, so it could’ve been the other way around so you know it’s a lot of it’s about attitude, and I think that transfers into appearance and how you deal with that (Paul).

In contrast, some interviewees felt like fraudsters because their injuries were less severe than others’. This created a sense of illegitimacy, as noted above in “Being seen as an injured war veteran”; instead of benefitting from downward comparison, participants felt guilty and ashamed.

When someone like me turns up and then there’s blokes who haven’t got any legs, you kind of feel a bit like oh, actually I’m a bit of a, a fraudster here (John).

### Active coping

As well as using cognitive strategies, many participants demonstrated activities, actions and behaviors that helped them cope; though not all were helpful for all participants. Five sub-themes represent active coping: Rebuilding self; Recovery via sporting activities; Creating safe narratives to manage others; Making it work for me; and, Avoidance.

#### Rebuilding self

The negative affect of injuries on self-esteem and self-worth was multifaceted, often relating to lost military identity, confidence, and the challenges of looking different. Striving for and achieving new successes and finding ways to make meaningful contributions, enabled over half of the participants to rebuild their sense of self, confidence, worth and esteem. Successes and activities included climbing mountains, public speaking, completing charity events, returning to education and re-qualifying in a new career, and being a representative of the injured veteran and disabled communities. Clothing was used as a new way to express themselves and to appear positive and confident.

I was proud of myself for I could’ve packed it in I could’ve said no this isn’t for me … but I pushed on with it, with their help and I got there and I managed to do it … . and that built my confidence massively, so that was quite a big leap for me … little bits of confidence building is the way to go (Paul).

Among those still serving, rebuilding physical ability and fitness to return to their serving roles seemed associated with confidence and self-worth. This was validated by other personnel who, upon noticing physical signs of injury, commended their ability to return to service.

I’ve just finished company command and at that people, you know, quite complementary, they asked questions and they’d say, ‘Oh it’s-, you know, inspiring to see, when you’ve had such significant injury that you’ve got yourself back up to fitness, to be able to achieve this.’ Which is, I mean it’s nice to hear (Daniel, serving).

#### Recovery via sporting activities

Ten participants, including one serving personnel, engaged in sports rehabilitation. This promoted physical recovery, rebuilt confidence; created focus and motivation; provided opportunities to feel part of a team; and, for some, created a stepping stone to new careers in sport or sport-related business. However, potential negative impacts of using sport in recovery were also raised. Some felt that relying heavily on sporting success to sustain a sense of worth could be risky and result in neglect of family or other important areas of life, including securing employment and establishing a “normal life.” It was suggested that elite sporting events with veterans contributed to a public perception that all injured veterans are elite athletes, which could be problematic for those not interested in sport, or unable to compete at an elite level; potentially leading to feelings of failure or low worth.

Getting involved in sport and adaptive sport actually it’s helped me kind of come to terms again you know come to terms with these scars and the injury and actually be a lot more comfortable with it (Henry).

So, you know real encouragement to go off and climb Mount Everest or cross the North Pole … or race across America, row the Atlantic, go to the Paralympic games … if you’re not doing those things then you do feel like a failure (Arron).

#### Creating safe narratives to manage others

Several participants found having prepared and “safe” narratives in response to unsolicited questions helpful, enabling them to respond clearly with minimal emotional demand, while providing enough information to satisfy the questioner. Narratives were also important in managing the impact of their appearance on children (their own and others’), through education, normalizing injuries, disability and looking different, and putting children at ease. In both the veterans’ personal narratives and the narratives used to manage the impact on children, humor often featured, especially in attempts to put others at ease.

You go well his job was to search for IEDs, yeah well you missed that one, you know and it’s funny, it’s just part, maybe that is just part of the coping (Charles).

If kids are looking I’ll say, “what do you think of the leg?”, and I’ll take it off and I’ll let them have a feel of it and, explain how I did it, and I get them to stand on one leg and balance as long as they can, and just to, you know (Alice).

#### Making it work for me

Five participants, including one serving, found assertive ways to make their changed appearance work for them. Some were assertive in managing uncomfortable social situations, for example, leaving events that made them feel bad, or being direct with people who made inappropriate comments. Some veterans described using “benefits” of being disabled and labeled as an injured veteran, by gaining sponsorship for sporting activities, writing and selling their stories, and priority and space when traveling. Ensuring they were physically and mentally fit to engage in certain social situations helped some veterans manage their lives. Consistent across these approaches was that they were taking control and being assertive.

If I’m in a situation where I don’t I’m not feeling comfortable and confident then I’ll ask myself “do I need to be here?” Is this important to me? No, okay let’s fuck off (Paul).

I try not to be out and about when I’m in pain. Sort of best not to be in the public when you’re when you’re suffering (Mark).

#### Avoidance

Avoidance as a coping strategy took two forms. Over half of the participants, including two serving personnel, made efforts to conceal, camouflage, or minimize their visible difference and changed appearance. In addition, four veterans used activity and keeping busy to avoid facing emotions related to their appearance and their injury more broadly.

Probably that’s why I didn’t wear shorts so often, because I don’t want people to see that (Dominic).

I think there’s a huge amount of fear in what happens when you stop running at hundred miles an hour coz then well you’ve got to contend with all this (Charles).

### Appearance support, delivery, and barriers

Veterans were asked directly about appearance specific support leading to four sub-themes representing their support experiences: Obstacles to acknowledging appearance; Appearance support we’d like; Barriers to care; and, Delivery preferences.

#### Obstacles to acknowledging appearance

The majority reported that appearance had rarely been discussed and did not recall being offered support for appearance-related issues. Physical recovery was prioritized and often appearance concerns arose only once physical recovery stabilized. Some reflected that earlier in their recover they had struggled with their changed appearance, but were unable to identify these feelings as related to their appearance at the time. Some felt that the military environment is not a safe place to talk about appearance and that appearance-related concerns may be interpreted as vanity. While many would have accepted appearance-support had it been offered, one veteran and one serving participant saw no benefit since “it would not change anything,” preferring instead to physically cover, conceal or minimize their scars.

Definitely, definitely, definitely, it was really important (appearance) at the time, I just couldn’t address it, couldn’t, couldn’t put a label on it (Benjamin).

#### Appearance concerns I’d like support with

Ten of the veterans identified specific appearance concerns they would like support with, including: ways to rebuild confidence; how to dress with “no legs”; adjusting to looking different; how to manage public attention; and how to communicate their injury to their children. Specific support for families was discussed, including emotional support to help them adjust to their family members’ changed appearance and information around how best to interact and communicate with them normally and sensitively.

Even just someone saying by the way when you go out people are going to stare and they’re going to ask you questions and they’re going to do this and this and this is how you’re going to feel when you wear certain clothes and you know almost like a life education workshop would have just been so valuable, and you know that almost automatically comes with ways in which you can solve the problem (Arron).

But not knowing what to tell a nine-year-old child (pause) it’s yeah you don’t want to mentally scar them as well, which obviously it does (Ethan).

#### Barriers to care

Seventeen participants, including two serving, expressed barriers to seeking support that are associated with the military culture of strength, hardiness, masculinity and self-reliance. These barriers seemed exacerbated among those still serving. Being perceived as weak for seeking support was reported, including references to appearance support being “pink and fluffy.” Concerns of weakness were heightened among those who considered their injury to be less severe, lacking legitimacy, and feeling like fraudsters. Those who were concerned about the legitimacy or severity of their injury or experiencing survivor guilt, felt undeserving of support. Career impact, trustworthiness of civilian care providers, the potential to “open cans of worms,” and only accessing support when it was directly offered, were additional barriers.

I felt guilty about using that stuff so I didn’t bother I didn’t engage with … I felt again because of the way I felt about myself I thought yeah do you know what, this isn’t for you, this is for people who’ve got bigger injuries (Henry).

I’m in an environment where if I ask for help, it shows a sign of weakness, it’s all crap but that’s the kind of thing that that goes around (Alice).

#### Delivery preferences

Six participants expressed preferences for support delivered by those with a lived experience of conflict injuries or at least of serving in the military, in a relaxed and informal environment and when the timing is “right,” though this differed between the veterans.

I don’t know he was just a nice guy and you’re sat there very casually there was nothing formal about it he came into the hospital I was still sitting there with bandages on and he’s chatting away to me (Paul).

## Discussion

Reflexive TA of 23 interviews identified six master themes representing the appearance-related challenges, military specific protective factors, coping skills and support needs of UK military personnel and veterans who sustained appearance-altering injuries. Difficult and intrusive social situations was the most common reported challenge. This is consistent with experiences of civilians with visible differences (Martin et al., 2017) and indicates a societal intolerance of appearance diversity. Evidence that social stigma may affect post-service employment is notable since it has been reported that veterans per se may experience employment discrimination (Keeling et al., 2018). Therefore, veterans with a visible difference may be at risk of experiencing an intersection of employment discrimination due to their veteran status and altered appearance. Concerns about the social impact on children highlights the wider impact of social stigma and indicates a need for support for children of injured personnel and veterans.

The impact of appearance-altering injuries on romantic relationships is important since they provide a key source of support, are central to identity, and are beneficial for health and wellbeing (Badr et al., 2011). The impact of appearance-altering injuries on romantic relationships is consistent with research conducted with the general population (Mathias & Harcourt, 2014; Sharratt et al., 2018). Challenges with mobility as well as body image concerns impacted sexual intimacy for some veterans in this study. This is consistent with experiences of men with spinal cord injuries and indicates that veterans might benefit from support that aims to help them develop new sexual repertoires (Warren et al., 2018).

Adding to Weaver et al.’s (2014) proposition, the current study provides evidence that body image dissatisfaction (BID) may be exacerbated by the military context where the physical body represents operational fitness, strength and military identity (Godfrey et al., 2012). This appears to have implications for body image, self-esteem and self-worth. Additional support for Weaver et al. (2014) is in the experiences of those whose changed appearance acted as reminders of their injury and associated trauma. This is consistent with general population research indicating a positive association between appearance concerns and trauma symptoms among individuals with burn injuries (Shepherd, 2015).

This study adds to existing evidence (Akyol et al., 2013; Weaver et al., 2014), that personnel and veterans with appearance-altering injuries may experience psychological distress related to how they feel about their changed bodies. This highlights an unmet support need for military veterans in the UK, and possibly other countries. In addition, disruption to the self-concept represented by the ability to recognize the self (Wisely & Gaskell, 2012) may indicate a specific support need, especially among those medically discharged who may experience compounding identity disruptions (Keeling, 2018).

Despite these challenges, resilience and coping were evident, including military specific factors, and some that differentiate the experience of deployment injuries versus training injuries. This has implications for support provision. Mostly, it should not be assumed that interventions developed for civilians will be effective for military populations. Moreover, those with training injuries may not benefit from the same protective factors as those with deployment injuries. In particular, recovery and rehabilitation with other injured personnel may be unique to the military. Civilian adults with spinal cord injuries report that a challenge to recovery was feeling socially disconnected from a supportive community of others with similar injuries who could relate to their specific experience (Engblom-Deglmann & Hamilton, 2020). This may also be true for those injured during training.

The assumption that the public draw on injured veterans’ visible differences to identify them as heroic and honorable, benefitted some veterans, alleviating concerns of being judged negatively. Injuries as a “badge of honour” is consistent with reports from injured female US veterans (Cater, 2012). However, not all veterans experience the public’s perception positively. Caddick et al. (2020) suggest that a “hierarchy of injuries” has been constructed through media reporting that frames military injuries as either “heroic” (combat) or “non-heroic” (non-combat/training). This study indicates that the perception of a hierarchy of injuries may impact recovery and adjustment among veterans with appearance-altering injuries.

Using cognitive coping approaches such as attaching meaning and re-framing experiences is consistent with US female veterans who found similar approaches important for positive adjustment to amputation (Cater, 2012). Having an optimistic disposition, reported in this and previous research with veterans (Cater, 2012), and the general population (Clarke et al., 2014), and self-compassion, found in this and previous research (Clarke et al., 2020), were found to aid coping. These are modifiable skills that could be effective components of interventions to support veterans with appearance-altering injuries. Engaging in discourses and cognitions that minimize the importance of appearance, such as focussing on functionality, have also been shown to be beneficial to manage body image concerns (Alleva et al., 2018) and indicates another targetable factor of appearance support for injured veterans.

Reporting that “it could have been worse” was also found among injured US female veterans (Cater, 2012) and in the general population (Egan et al., 2011). As an extension of social comparison theory, making downward comparisons can elevate self-regard (Jefferies et al., 2018) and facilitate normalization of injury-associated difficulties, which can allow the individual to persevere with “normal” activities. For those who considered their injuries less severe or low on the hierarchy of injuries, making downward comparisons instead led to feelings of guilt and shame. Downward comparisons may only be effective for individuals who perceive their injury as legitimate and worthy of comparison.

Engaging in activities to rebuild confidence and self-worth has been found among other UK (Caddick et al., 2020) and US (Cater, 2012) veterans and provides additional evidence that active coping may help adjustment with appearance-related difficulties, consistent with evidence from civilian amputees (Wetterhahn et al., 2002). The use of narratives to cope with difficult social situations fits with evidence of the benefits of social skills training (Zucchelli et al., 2018), which can be a beneficial component of supportive interventions for people with a visible difference (Norman & Moss, 2014). While humor can be a useful strategy, it can also be used to avoid difficult emotions which could maintain rather than alleviate distress (Zucchelli et al., 2020). Avoidance in the form of concealing scars and other appearance-fixing tendencies (Cash et al., 2005) may also be counterproductive. While such behaviors may minimize distress in that moment, long-term they may maintain rather than alleviate appearance-related distress, avoiding distressing emotions rather than addressing them.

While personnel and veterans found ways to cope, many would welcome appearance specific support. The timing of appearance support being offered is important, with physical recovery likely prioritized early in recovery and rehabilitation. Additional obstacles to acknowledging appearance difficulties and accessing support may be related to military culture which has been described as synonymous with masculinity (Morgan, 1994). This may manifest as concerns about being perceived as vain, as well as concerns of being perceived as weak. This finding, alongside the support preferences, are consistent with previous research with veterans (Keeling et al., 2017), including a preference for support being delivered by other veterans, preferably those who have been injured or deployed, and should be considered when planning support.

### Strengths and limitations

This study is the first to investigate body image, psychosocial and appearance-related experiences of UK military personnel and veterans with appearance-altering injuries. The specific experiences of military populations have, until now, been omitted from the visible difference literature, and this study adds to a small but growing international literature examining body image among the military. Participants were self-selecting, so the results should be interpreted considering possible biases of a sample who opt in to talk about their experiences, especially considering barriers to acknowledging and discussing appearance within the military. The female perspective is not fully represented, limiting the extent to which conclusions about females can be made. This is also true for serving personnel. The sample is majority white British and not representative of injured personnel of other ethnicities. Since this is a qualitative reflexive TA study, the aim is not to generalize to the wider population but to gain in-depth understanding of a sample with a shared experience. Therefore, findings and implications should be considered in this epistemological and ontological frame.

### Implications

This study indicates a need for appearance specific support for military veterans who sustained appearance-altering injuries. While there are similarities between the experiences of military populations and civilians with a visible difference, evidence highlights military specific factors that may affect recovery and adjustment. This requires further quantitative investigation to compare these populations. In addition, this novel research highlights several appearance specific challenges that require further research to better understand possible areas requiring targeted support. This includes the intersection of employment discrimination and the impact on post-service civilian employment, the impact of appearance-related social stigma on children, and the impact of appearance-altering injuries on romantic relationships and intimacy.

Changes to appearance acting as a reminder of trauma requires additional investigation and indicates that appearance support for injured veterans be trauma informed. Future support for veterans with appearance-altering injuries must consider barriers and delivery preferences, including gaining a better understanding of a hierarchy of injuries and its impact on psychological wellbeing and help seeking.

## Conclusions

Thematic analysis of interviews conducted with military personnel and veterans who sustained appearance-altering injuries indicates that in the context of the broader recovery experience, military personnel and veterans experience a variety of challenges related to their changed appearance and body image. While many of these experiences are consistent with civilians who have sustained burn injuries and limb-loss, nuances in the challenges, protective experiences, coping approaches, and barriers and preferences for support are evident. These differences indicate that military populations with appearance-altering injures may require tailored support to help them manage and adjust to any appearance challenges and body image concerns.

## Data Availability

Due to the qualitative nature of this research and difficulties fully anonymising data, participants did not agree for their data to be shared publicly, so supporting data is not available.
